# Expression and clinical significance of matrix metalloproteinase-17 and -25 in gastric cancer

**DOI:** 10.3892/ol.2014.2747

**Published:** 2014-12-01

**Authors:** YING WANG, SHI-JIE YU, YAN-XIA LI, HE-SHENG LUO

**Affiliations:** Department of Gastroenterology, Renmin Hospital of Wuhan University, Wuhan, Hubei 430060, P.R. China

**Keywords:** gastric cancer, matrix metalloproteinase 17, matrix metalloproteinases 25, immunohistochemistry, reverse transcription-quantitative polymerase chain reaction

## Abstract

The aim of the present study was to investigate the expression and clinicopathological features of matrix metalloproteinase 17 (MMP17; also known as MT4-MMP) and MMP25 (also known as MT6-MMP) in gastric cancer. Immunohistochemistry and reverse transcription-quantitative polymerase chain reaction were used to detect the expression of MMP17 and MMP25 in 42 cases of gastric carcinoma and normal tissues, and 40 cases of atrophic gastritis. The expression of MMP17 in the normal gastric and atrophic gastritis tissues was significantly lower than that in the gastric cancer tissues (P<0.05). The expression of MMP25 in the gastric cancer and atrophic gastritis tissues was markedly higher compared with the normal gastric tissues (P<0.05). The expression of MMP17 and MMP25 was significantly associated with the depth of tumor invasion, lymph node metastasis and serous membrane involvement (P<0.05), but not with patient age and gender, or lesion length, site and histological grade (P>0.05). Therefore, this indicates that the expression of MMP17 and MMP25 is increased with the degree of progress of gastric carcinoma. The detection of MMP17 and MMP25 expression may have clinical value in predicting the prognosis of patients with gastric cancer.

## Introduction

After lung cancer, gastric cancer is the second most common cause of cancer-associated mortalities worldwide (1). Despite an overall decline in the incidence of gastric cancer, the disease remains prevalent in Asian countries (1,2). At present, the majority of patients with gastric cancer are diagnosed with late-stage disease, which unlike the curable early stages, has limited therapeutic strategies (3). Currently, surgery and combination chemotherapies confer an overall five-year survival rate of <24% for patients with advanced gastric cancer (4,5). Therefore, an understanding of the molecular and genetic factors that underlie the progression of gastric cancer may enable the identification of novel gastric biomarkers and potential targeted therapies.

Prior to metastasizing, tumor cells must complete a multi-step progression, which includes detachment, local invasion and motility. Throughout these stages, causative molecules, including matrix degradation enzymes, can be used as prognostic factors (6). The matrix metalloproteinases (MMPs) are a family of enzymes located in the extracellular milieu of various tissues, and with important roles in extracellular matrix degradation and angiogenesis during tumor invasion and metastasis. The overexpression of MMPs can promote tumor cell detachment and metastasis, which have been associated with malignancy and a poor clinical outcome for patients (7,8). At present, there are 26 known MMPs, which share a number of common structural and functional similarities, but differ in their substrate specificity (9).

MMP-17 (also known as MT4-MMP) and MMP25 (also known as MT6-MMP) are held in the plasma membrane by a glycosyl-phosphatidyl inositol (GPI) anchor, which equips the enzymes with a set of regulatory and functional mechanisms that differentiates these subtypes from other members of the MMP family. Recent studies have demonstrated that GPI-membrane type (MT)-MMPs are highly expressed in human cancers (10), where they have a role in disease progression. Furthermore, biochemical and functional evidence also highlights the distinct properties of the enzymes. The present study investigated the expression and clinicopathological features of GPI-MT-MMPs in gastric cancer.

## Materials and methods

### Tissue samples

In total, 42 tissue samples were obtained from patients with gastric cancer who had undergone surgery, with no radiotherapy or chemotherapy, between January 2011 and December 2013, in the Renmin Hospital of Wuhan University (Wuhan, Hubei, China). The study was approved by the ethics committee of Renmin Hospital of Wuhan University and written informed consent was obtained from the patients or the family of the patient. Subsequent to a physical examination, 42 subjects with normal gastric mucosa and 40 cases of atrophic gastritis were also enrolled in the study. Of all the tissue samples taken, one sample from each subject was immediately fixed in 4% paraformaldehyde solution and embedded in paraffin for immunohistochemical staining, while another was stored at −80°C for reverse transcription-quantitative polymerase chain reaction (RT-qPCR) testing.

### Immunohistological analysis

In total, 4-μm thick sections of the tissue arrays were deparaffinized, and antigen retrieval was performed by microwaving the slides in 7.5 mM sodium citrate buffer (pH 6.0; Beyotime Institute of Biotechnology, Shanghai, China). Subsequent to rinsing in Tris-buffered saline (TBS; pH 8.0; Beyotime Institute of Biotechnology), endogenous peroxidase activity and non-specific background staining were blocked by incubating the samples for 30 min in 3% hydrogen peroxide (Beyotime Institute of Biotechnology) in methanol, followed by 30 min in 0.3% bovine serum albumin in TBS. The slides were then rinsed for 2 min each in TBS, TBS containing 0.01% Triton X-100 (Beyotime Institute of Biotechnology), and then TBS again. Next, the slides were incubated for 1 h at room temperature with rabbit antiserum pAb107 to human MT4-MMP and MT6-MMP at a dilution of 1:400 (Santa Cruz Biotechnology, Inc., Santa Cruz, CA, USA). The slides were then rinsed in TBS and incubated for 30 min with goat anti-rabbit immunoglobulin G conjugated to a horseradish peroxidase-labeled polymer (EnVision+ System; Dako, Glostrup, Denmark). Incubation was followed by additional TBS rinses, visualization with diaminobenzidine chromogen and counterstaining with hematoxylin. The negative controls were treated with pre-immune rabbit serum instead of the primary antibody. The sections were analyzed for histopathological features by a pathologist blinded to the patient data. The cell count was obtained using a microscope (magnification, ×400), and five fields were randomly selected for each slice, with each specimen represented by three slices. The expression of MMP-17 and -25 was identified by the percentage of positive cells and the staining intensity scores. The percentage of positive cells was ranked according to four grades: i) ≤5%, 0 points; ii) 6–25%, 1 point; iii) 26–50%, 2 points; and iv) >50%, 3 points. The staining intensity scoring criteria was as follows: i) no staining, 0 points; ii) weak staining (pale-yellow), 1 point; iii) moderate staining (brown), 2 points and; iv) strong staining (brown), 3 points. The sum of the two ratings was scored semi-quantitatively from zero to six as follows: 0, negative; 1–2, weak staining; 3–4, moderate staining; and 5–6, strong staining. For the negative control group, phosphate-buffered saline was used as an alternative to the primary antibody.

### RT-qPCR

The total RNA was extracted from the gastric carcinoma, atrophic gastritis and normal gastric tissues with TRIzol reagent (Invitrogen Life Technologies, Carlsbad, CA, USA), and cDNA synthesis was performed using the Transcriptor First Stand cDNA Synthesis kit (Roche, Basel, Switzerland) using 2 μg total RNA. The RT-qPCR was performed with the LightCycler 480 SYBR Green I Master (Roche) using the LightCycler 480 Real-Time PCR System, according to the manufacturer’s instructions (Roche). Using the published cDNA sequence (GenBank Accession no. AF219624), primers were designed to amplify a product of human MMP17 [forward, 5′-GGT GCG TGC ACT CAT GTA CT-3′; and antisense, 5′-TCA TCG TCA AAG TGG GTG TC-3′ (product length, 216 bp)], MMP-25 [forward, 5′-CCC AAA CCC CAT ATG ACA AG-3′; and antisense 5′-GGG GCC TTT GAA GAAGAA AG-3′ (product length, 164 bp)] and β-actin [forward, 5′-CAC GAT GGA GGG GCC GGA CTC ATC-3′; and antisense, 5′-TAA AGA CCT CTA TGC CAA CAC AGT-3′ (product length, 240 bp)]. The following PCR conditions were used: Initial denaturation at 95°C for 10 sec, denaturation for 40 cycles at 95°C for 10 sec, annealing at 60°C for 10 sec and extension at 72°C for 20 sec. The relative expression levels of mRNA were calculated as ratios to the reference gene, β-actin.

### Statistical analysis

The data are presented as the mean ± standard error of the mean. χ^2^ test was used to analyze the clinical and pathological characterstics of the patients. The statistical significance between groups was determined using a two-tailed Student’s t-test or one-way analysis of variance. χ^2^ and t: the comparation of normal gastric tissue and atrophic gastritis; χ_1_^2^ and t_1_: the comparation of normal gastric tissue and gastric cancer; χ_2_^2^ and t_2_: the comparation of gastric cancer and atrophic gastritis. P<0.05 was considered to indicate a statistically significant difference.

## Results

### Stage, grade and location of gastric cancer

In total, 124 patients with gastric cancer, atrophic gastritis or normal gastric tissues were included in the present study. The mean age of the patients was 54 years, and 67% of the participants were male. According to the TNM classification of malignant tumors developed by the American Joint Committee on Cancer Classification, the Japanese Gastric Cancer Research and the International Union Against Cancer (11), the stages, histological grade and location of the 42 cases of gastric cancer were as follows: i) T1, 18 cases; T2–T4, 24 cases; N0, 22 cases; and Nl–N3, 20 cases; ii) grade I, 13 cases; and grade II–III, 29 cases; and iii) antrum, 16 cases; the gastric body, 19 cases; and the gastric cardia, 19 cases.

### Expression of MMP17 in gastric cancer, atrophic gastritis and normal gastric tissues

**Figure 1:**
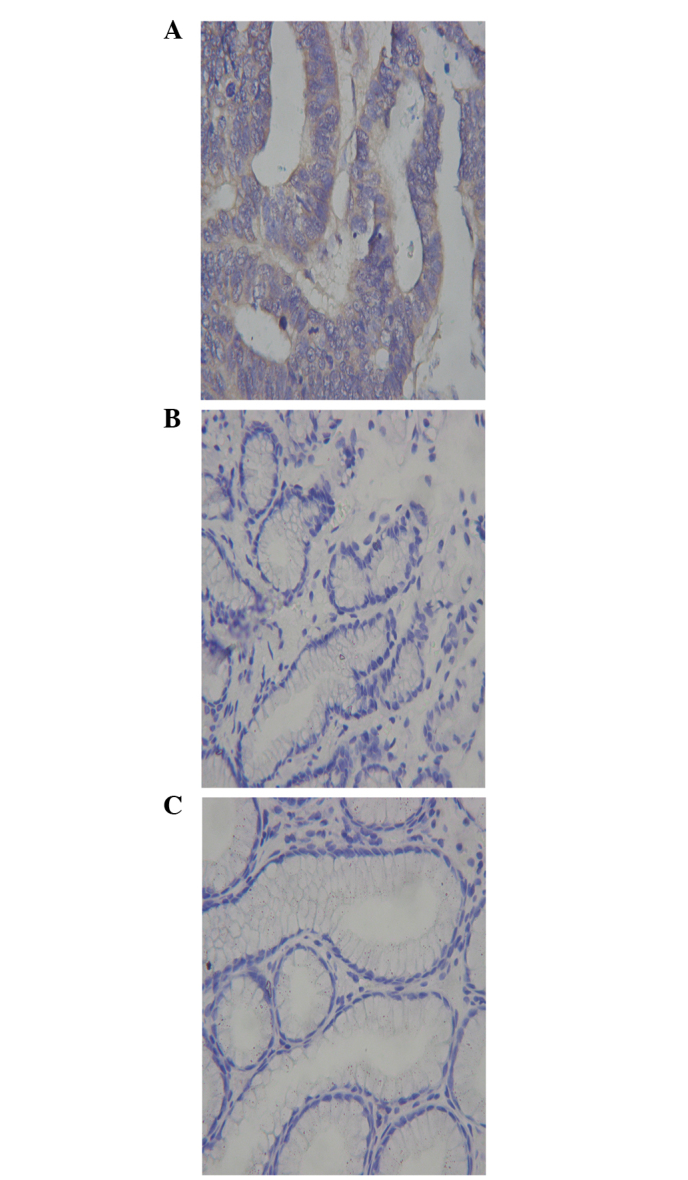
Immunostaining for the expression of matrix metalloproteinase 17 in (A) gastric cancer, (B) atrophic gastritis and (C) normal tissue (magnification, ×400).

**Figure 2:**
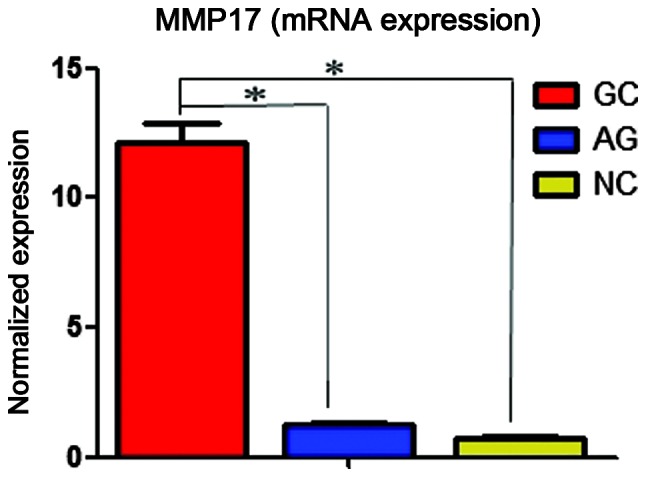
Expression of matrix metalloproteinase 17 mRNA in gastric cancer (GC), atrophic gastritis (AG) and normal tissue (NC). ^*^ P<0.05.

The expression of MMP17 protein in the cytoplasm was identified by pale-yellow, brown or tan staining. The MMP17-positive cells had a scattered or nest-like distribution in the gastric cancer tissues, and were markedly expressed on the edge of the cancer nest (Fig. 1A). In addition to the cancer cells, MMP17-positive staining was also observed in nearby cancer stromal cells, which suggested that stromal cells have an important role in the process of tumor invasion and metastasis. The expression of MMP17 in atrophic gastritis and normal gastric tissues is presented in Fig. 1B and C. No significant difference was identified between the expression of MMP17 in the normal tissue and atrophic gastritis specimens (3/42 and 4/40 cases, respectively; χ^2^=0.21; P>0.05). However, the expression of MMP17 in the gastric cancer specimens was significantly higher than that in the normal and atrophic gastritis tissues (31/42 cases; χ_1_^2^=38.74; χ_2_^2^=34.10; P<0.05). No significant difference was identified between the mRNA expression of MMP17 in the normal gastric and atrophic gastritis tissues (0.754±0.074 and 1.226±0.082, respectively; t=0.602; P>0.05), however, an evident difference was observed in the gastric cancer tissues (12.126±0.743; t_1_ 8.079; t_2_=4.493; all P<0.05) (Fig. 2).

### Expression of MMP25 in gastric cancer, atrophic gastritis and normal gastric tissues

**Figure 3:**
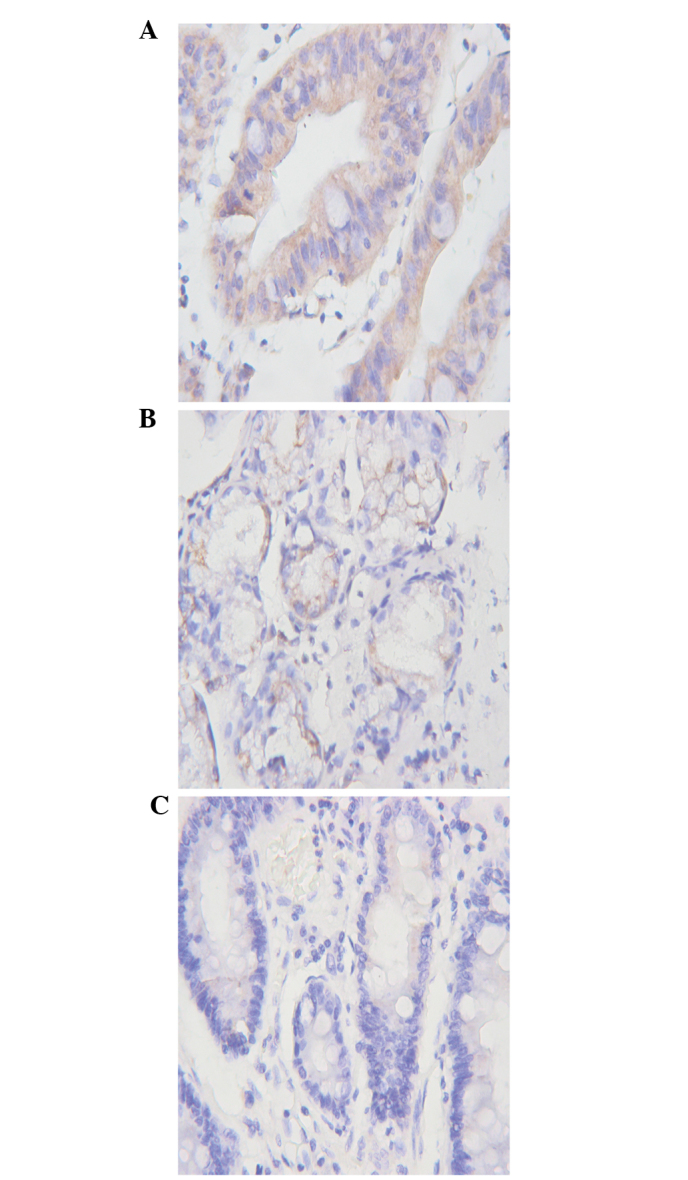
Immunostaining for the expression of matrix metalloproteinase 25 in (A) gastric cancer, (B) atrophic gastritis and (C) normal tissue (magnification, ×400).

**Figure 4:**
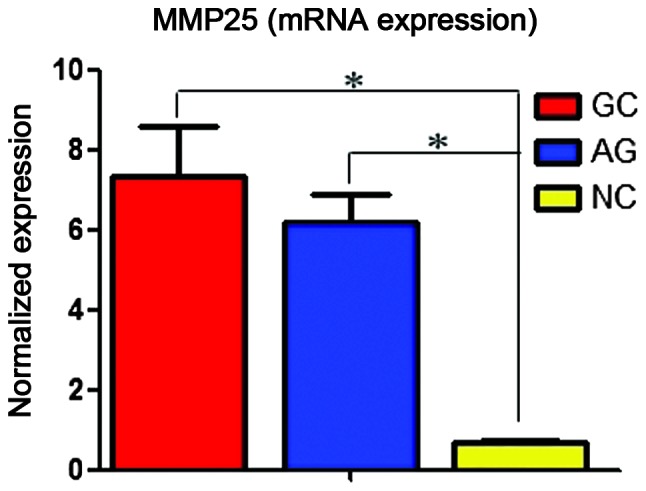
Expression of matrix metalloproteinase 25 mRNA in gastric cancer (GC), atrophic gastritis (AG) and normal tissue (NC). ^*^P<0.05.

MMP25 was expressed in the normal gastric, atrophic gastritis and gastric carcinoma tissues. However, MMP25-positive staining was significantly higher in the gastric cancer and atrophic gastritis tissues (40/42 and 33/40 cases, respectively), than in the normal gastric tissues (9/42 cases; χ_1_^2^=44.08; χ^2^=28.19; P<0.05). Furthermore, no significant difference was identified between the expression of MMP25 in the atrophic gastritis and gastric cancer tissues (χ_2_^2^=2.223; P>0.05) (Fig. 3). The expression of MMP25 mRNA in the normal gastric tissues was significantly lower than that in the atrophic gastritis and gastric carcinoma tissues (0.703±0.014, 6.175±0.702 and 7.328±1.235, respectively; t=7.149, t_1_=6.123; P>0.05). In addition, no significant difference was identified between the atrophic gastritis and gastric carcinoma tissues (t_2_=0.602; P>0.05) (Fig. 4).

### MMP17 and clinicopathological features

The present study demonstrated that MMP17 protein and mRNA expression was associated with the depth of tumor invasion, lymph node metastasis and serosal involvement of the gastric cancer patients (P<0.05), but not with age, gender, lesion length or histological grade (P>0.05; Table I). The expression of MMP17 in advanced gastric carcinoma was revealed to be higher than that in early-stage disease (21/24 and 10/18 cases; t=2.437; P<0.05). Furthermore, it was identified that MMP17 expression was elevated in patients with lymph node metastasis and serosal involvement.

### MMP25 and clinicopathological features

No significant difference in the expression of MMP25 between advanced gastric carcinoma and early-stage disease (17/20 and 19/22 cases, t=0.101; P>0.05) was identified. The MMP25 protein and mRNA expression was associated with the depth of tumor invasion, lymph node metastasis and serosal involvement of the gastric cancer patients (P<0.05), but not with age, gender, lesion length or histological grade (P>0.05; Table I).

## Discussion

The present study compared the expression of MMP17 and MMP25 in gastric carcinoma, atrophic gastritis and normal gastric tissues. The expression of MMP17 in the normal gastric and atrophic gastritis tissues was significantly lower than that observed in the gastric cancer tissues. MT-MMPs are efficient, pericellular, proteolytic enzymes that are presented at the cell surface by membrane anchoring domains. MMP-17 and MMP-25 are attached to the plasma membrane via a GPI anchor. This equips the enzymes with distinct functional and regulatory properties that distinguish MMP-17 and MMP-25 from other members of the MT-MMP subfamily. Despite their discovery almost a decade ago, studies conducted on GPI-MT-MMPs are limited compared with other MT-MMPs. However, recent evidence (12–14) has revealed that GPI-MT-MMP expression is elevated in human cancers. The data from the present study demonstrated that the GPI-MT-MMPs MMP17 and MMT25, similar to other MT-MMPs, are highly expressed in gastric carcinoma. In addition, the fact that MMP25 is highly expressed in atrophic gastritis suggests that it may be involved in the early stage of tumor development. The variety of physical properties of GPI-MT-MMPs encourages further study to determine their involvement in the development of tumors.

The clinicopathological features were closely associated with the prognosis of cancer (15,16). The association between the expression of MMP17 and MMP25 and disease clinicopathological features was investigated in the present study. It was identified that the expression of MMP17 and MMP25 was significantly associated with the depth of tumor invasion, lymph node metastasis and serous membrane involvement, but not with patient age and gender, or lesion length, site and histological grade. This observation was in accordance with other MMPs (17–20). Since the depth of tumor invasion, lymph node metastasis and serous membrane involvement were closely associated with tumor progression, MMP17 and MMP25 were associated with tumor progression. Furthermore, it was demonstrated that GPI-MT-MMPs, in addition to other MT-MMPs, play a significant role in tumor progression. However, their contribution to the development of gastric carcinoma is unclear, and requires further investigation.

In conclusion, the expression of MMP17 and MMP25 was increased in the gastric cancer tissues in the present study. Furthermore, the detection of MMP17 may be of clinical value for the prognosis of patients with gastric cancer. The present study included a limited number of cases and was a single-center study. Therefore, further analysis is required to determine whether MMP17 expression in gastric cancer exhibits regional differences. In addition, as gastric cancer is a multi-factorial and multi-linkage disease, the specific role of MMP17 in disease progression warrants further investigation.

## Figures and Tables

**Table I:** Association between matrix metalloproteinase-17 and -25 protein and mRNA expression and clinical pathological characteristics.

Clinical pathological features	n	MMP17 protein			P-value	MMP17 mRNA	P-value	MMP25 protein			P-value	MMP25 mRNA	P-value
	
		−	+	%				−	+	%			
Age, years													
≤60	23	6	17	73.91		0.483±0.031		5	18	78.26		0.304±0.003	
>60	19	5	14	73.78	0.987	0.469±0.029	0.209	1	18	94.74	0.205	0.311±0.007	0.288
Gender													
Male	25	6	19	76.00		0.484±0.030		5	20	80.00		0.346±0.005	
Female	17	5	12	70.59	0.699	0.472±0.028	0.527	1	16	94.12	0.263	0.370±0.001	0.312
Size, cm													
≤2	20	6	14	70.00		0.482±0.034		3	17	85.00		0.315±0.003	
>2	22	5	17	77.27	0.597	0.470±0.037	0.312	3	19	86.36	0.256	0.308±0.002	0.114
Depth of invasion													
T1	18	8	10	55.56		0.398±0.029		1	17	94.44		0.309±0.009	
T2–T4	24	3	21	87.50	0.021	0.513±0.043	0.009	5	19	79.17	0.986	0.536±0.002	0.006
Histological grading													
I	20	6	14	70.00		0.477±0.028		3	17	85.00		0.426±0.006	
II–III	22	5	17	77.27	0.597	0.529±0.038	0.195	3	19	86.36	0.256	0.418±0.005	0.102
Lymph node metastasis													
N0	22	9	13	59.09		0.403±0.035		5	17	77.27		0.337±0.002	
N1–N3	20	2	18	90.00	0.025	0.523±0.022	0.010	1	19	95.00	0.011	0.552±0.004	0.003
Location													
Antral	16	4	12	75.00		0.480±0.041		2	14	87.50		0.478±0.014	
Body	19	6	13	68.42		0.469±0.032		3	16	84.21		0.435±0.005	
Cardia	7	1	6	85.71	0.756	0.486±0.034	0.691	1	6	85.71	0.454	0.426±0.006	0.214
Serosal involvement													
No	19	8	11	57.89		0.432±0.034		5	14	73.68		0.326±0.017	
Yes	23	3	20	86.96	0.035	0.512±0.038	0.029	1	22	95.65	0.001	0.488±0.015	0.043
Survival time, years													
<2	10	7	3	30.00		0.490±0.031		5	5	50.00		0.326±0.075	
≥2	32	4	28	87.50	0.005	0.522±0.028	0.018	1	31	96.88	0.001	0.518±0.023	0.001

MMP, matrix metalloproteinase.
